# 2022 Mpox (monkeypox) outbreak: a concise review focused on new features of dermatological lesions^☆^

**DOI:** 10.1016/j.abd.2023.02.001

**Published:** 2023-03-17

**Authors:** Elena Lucía Pinto-Pulido, Miriam Fernández-Parrado, Francisco José Rodríguez-Cuadrado

**Affiliations:** a Department of Dermatology, Hospital Universitario Príncipe de Asturias, Universidad de Alcalá, Madrid, Spain; b Department of Dermatology, Hospital Universitario de Navarra, Pamplona, Spain; c Department of Dermatology, Hospital Universitario Puerta de Hierro, Madrid, Spain

Dear Editor,

Since May 2022, more than 84.000 monkeypox or mpox, confirmed cases have been reported from 110 different countries. Previously, mpox was confined to West and Central Africa, with few cases outside this region, always epidemiologically connected with African countries.^1^ As well as epidemiological changes, clinical differences have also been reported with respect to the typical clinical picture previously described in endemic countries.

We collected clinical and epidemiologic 2022 mpox data from the World Health Organization (WHO) official website and from PubMed-indexed articles published between June 2022 and January 2023. We selected case series of at least 185 patients which included detailed descriptions of dermatologic findings. We also included studies focusing on the histologic, sonographic, dermatoscopic, and genetic features.

According to data provided by the WHO, 96.6% of 2022 cases are men and among them, 84.2% were Men who have Sex with Men (MSM). Sexual relations were the most reported mechanism of transmission (69.9%) and party setting with sexual contacts was the most likely exposure category (46.8%).^1^ The proportion of MSM infection in large case series is even higher (99.8%‒100% men and 98%‒99% MSM), with an HIV positivity of 35.5%‒42%. Between 29% and 76% had a sexually transmitted concurrent infection.^2^, ^4^ These data, together with the preference of mucocutaneous lesions to settle in genital and perianal areas suggest contact during sexual intercourse as the main transmission mechanism.^2^ Before May 2022, preference for MSM had not been observed. Previously recognized mechanisms of transmission were contact with an infected animal or with respiratory droplets, body fluids, mucosa, skin lesions, and fomites from an infected person.^5^

Classic mpox clinical picture described before this outbreak began with a prodromal phase with fever, malaise, and lymphadenopathy. Afterward, a skin rash appeared, characterized by the sequential synchronous evolution of lesions from macules to papules, vesicles, and pustules.^5^ A high number of skin lesions was described (mean of 370 in a study from the 2007‒2011 Democratic Republic of the Congo outbreak).^3^ According to large 2022 case series reports, the prodromal period was only present in about half of the patients (36%‒61.5%), whereas the rest of them had no systemic symptoms or developed them after or at the same time as skin lesions. Fever was present in 54%‒62% and lymphadenopathy in 56%‒57.9%. Preferred localization for mucocutaneous lesions was genital or genitoanal area (53%‒56.4% genital, 34%‒41.6% perianal, 73%‒88.3% genital and/or perianal lesions).^2^, ^3^, ^4^ A median of 5 lesions was reported^3^ with less than 20‒25 lesions in the majority of patients (85%‒93%).^2^, ^4^

A significant number of patients (35.5%‒47%) showed cutaneous lesions at different stages.^2^, ^3^ Catala et al. described lesions in the likely inoculation areas as whitish papules that simulate pustules (pseudopustules), with a necrotic center (Fig. 1). They are not true pustules as they have solid content instead of liquid purulent content.^2^ Ultrasound descriptions support this appreciation as they do not show liquid but solid inflammatory lesions with marked intralesional vascularity (Fig. 2).^6^ Dermatoscopic features of these pseudopustules consist of a reddish-crusted hemorrhagic center with a whitish ring surrounded by an erythematous area (Fig. 3).^7^ Histologically, the aforementioned lesions are characterized by full-thickness epidermal necrosis, keratinocyte ballooning and eosinophilic cytoplasmic inclusions (Guarnieri’s inclusion bodies), together with a significant predominantly lymphocytic inflammatory infiltrate (Fig. 4).^8^ After these initial lesions, an eruption of vesicular and true small pustular lesions can occur, while macular rashes are rarer.^2^ WHO has reported 81 deaths,^1^ considerably less than previously reported mortality of 3.3%‒10.6%.^5^

**Figure 1:**
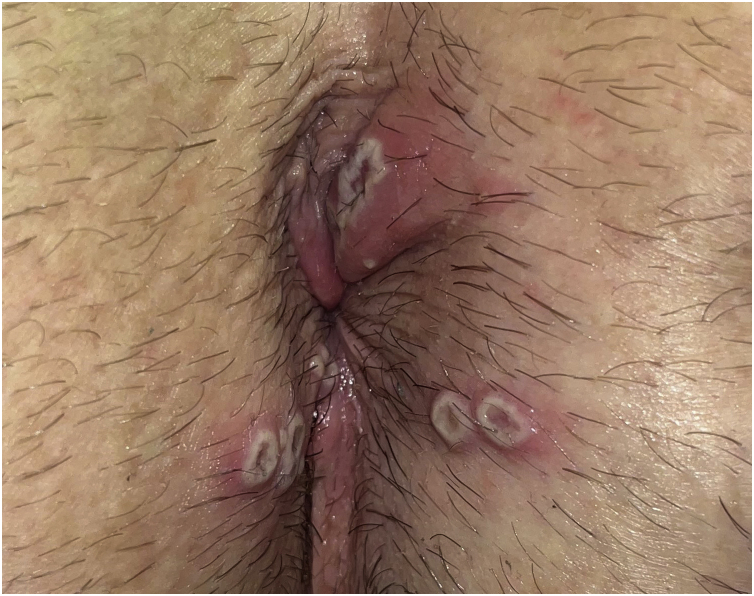
Clinical image. Perianal whitish papules with a necrotic center (pseudopustules).

**Figure 2:**
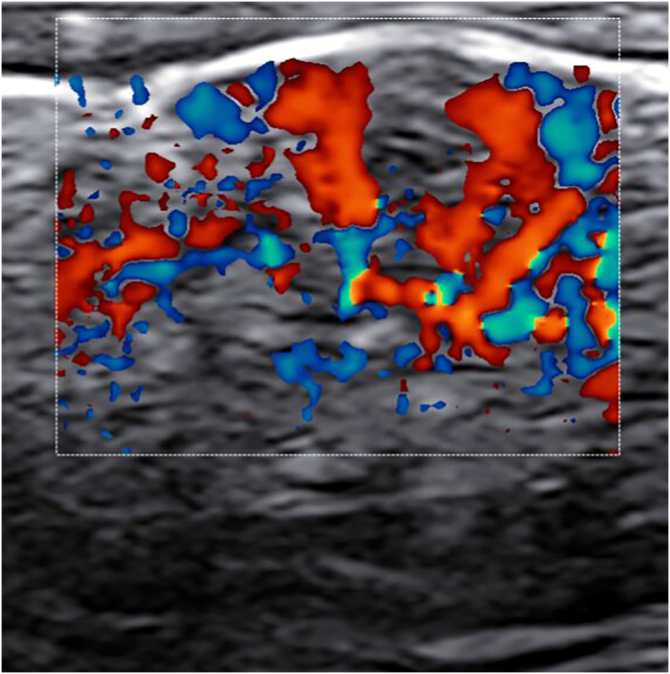
Ultrasound findings (22 Hz). Color-Doppler of a pseudopustule that highlights the marked intralesional vascularization, together with dermo-epidermal thickening and focal dermo-hypodermal hypoechogenicity.

**Figure 3:**
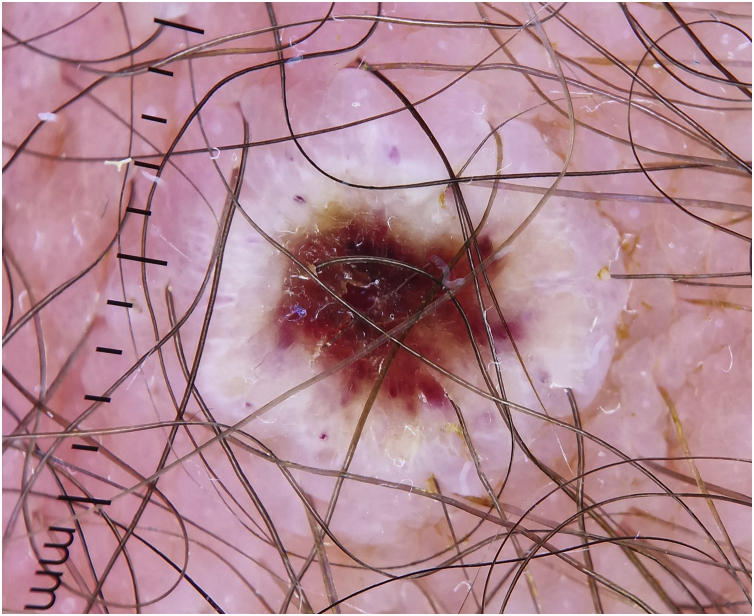
Dermatoscopic image. Pubic pseudopustule with reddish crusted center surrounded by a whitish ring.

**Figure 4:**
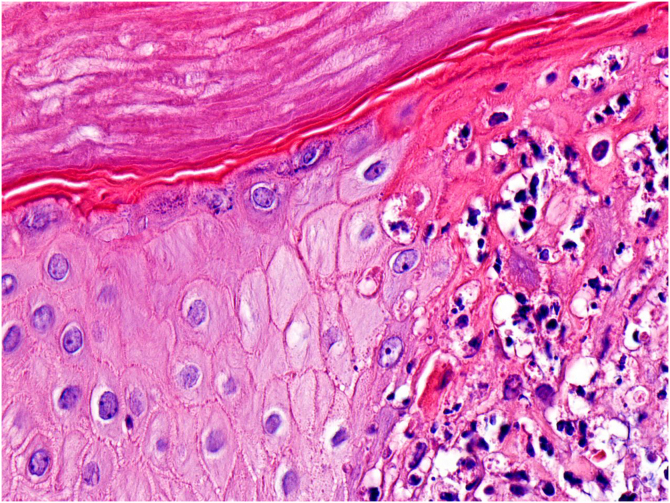
Histologic features (Hematoxylin & eosin, ×400). Epidermal keratinocytes with balloon cell degeneration together with a predominantly lymphocytic inflammatory infiltrate at the edges of the lesion.

Genome sequencing indicates that the monkeypox virus responsible for the 2022 outbreak corresponds to clade IIb (West African clade) and has phylogenetic linkage with previous 2018‒2019 cases. However, it constitutes a distinct phylogenetic branch (lineage B.1) with a higher number of genetic mutations than expected for Orthopoxviruses.^9^ This could be a concern in terms of immunization after suffering the disease and vaccination efficacy in future outbreaks. However, based on the course of the outbreak with a quick decrease in the number of cases, it appears that the smallpox vaccine provides cross-immunity, as theoretical studies had already suggested.^10^

In conclusion, as well as epidemiological changes, clinical differences have also been reported with respect to the clinical picture previously described in endemic countries. It remains to be determined whether these changes are due to virus genetic mutations, a different mechanism of transmission, distinct baseline characteristics of affected patients compared to those of endemic countries, or, more likely, by a combination of all these factors. Detailed dermatological description of lesions is essential for an accurate diagnosis since pseudopustules with a necrotic-hemorrhagic center are infrequent in other dermatological disorders.

## Financial support

None declared.

## Authors' contributions

Elena Lucía Pinto-Pulido: Approval of the final version of the manuscript; critical literature review; data collection, analysis, and interpretation; effective participation in research orientation; preparation and writing of the manuscript; study conception and planning.

Miriam Fernández-Parrado: Approval of the final version of the manuscript; critical literature review; data collection, analysis, and interpretation; effective participation in research orientation; manuscript critical review; study conception and planning.

Francisco José Rodríguez-Cuadrado: Approval of the final version of the manuscript; critical literature review; data collection, analysis, and interpretation; effective participation in research orientation; manuscript critical review; study conception and planning.

## Conflicts of interest

None declared.
